# NK Cell Levels Correlate with Disease Activity in Patients with Multiple Sclerosis on Ocrelizumab/Rituximab Therapy

**DOI:** 10.3390/ph17020150

**Published:** 2024-01-23

**Authors:** Simone Dal Bello, Simone Lorenzut, Emma Saccomano, Yan Tereshko, Gian Luigi Gigli, Carlo Ennio Pucillo, Mariarosaria Valente

**Affiliations:** 1 Clinical Neurology Unit, Santa Maria della Misericordia University Hospital, 33100 Udine, Italy; 2 Neurology Unit, Santa Maria della Misericordia University Hospital, 33100 Udine, Italy; 3 Neurology Unit, Department of Medicine (DMED), University of Udine, 33100 Udine, Italy; 4 Immunology, Department of Medicine (DMED), University of Udine, 33100 Udine, Italy

**Keywords:** multiple sclerosis, Ocrelizumab, Rituximab, anti-CD20, NK cells

## Abstract

Background: Recently, research on the pathogenesis of multiple sclerosis (MS) has focused on the role of B lymphocytes and the possibility of using specific drugs, such as Ocrelizumab and Rituximab, directed toward these cells to reduce inflammation and to slow disease progression. Objective: We aimed to evaluate the effect of Ocrelizumab/Rituximab on laboratory immune parameters and identify the predictors of treatment responses. Methods: A retrospective single-center study was conducted among patients who received infusion therapy with an anti-CD20 drug to treat MS. Results: A total of 64 patients met the inclusion criteria, with 277 total cycles of therapy studied. Compared with the baseline values, anti-CD20 infusions resulted in absolute-value and percentage decreases in B lymphocyte levels and increased the absolute and percentage levels of NK cells 3 and 5 months after therapy (*p* < 0.001). After multivariate logistic regression analysis, a reduced percentage level of NK cells 3 months after infusion could predict disease activity 6 months after Ocrelizumab/Rituximab administration (*p* = 0.041). Conclusions: Lower percentage levels of NK cells 3 months after anti-CD20 infusion correlate with the presence of disease activity 6 months after therapy, confirming a possible protective role of NK cells in MS.

## 1. Introduction

Multiple sclerosis (MS) is a chronic autoimmune inflammatory disease of the central nervous system (CNS), and it has important epidemiological and social impacts: with a prevalence of 50–300 affected persons per a population of 100,000, it is considered the leading cause of neurological disability in young people [1]. Neuropathologically, it is characterized by three distinctive features: neuroinflammation, demyelination, and axonal degeneration. Despite recent advances, the pathogenesis of MS remains unclear in many aspects. The leading promoters of the autoimmune response were considered the T lymphocytes; however, in recent years, great attention has focused on the role of B lymphocytes and on how, by acting on these cells with specific drugs, a containment of inflammation and a slowing down of the progression of the disease could be obtained [2]. Unsurprisingly, the most widely used second-line therapies for multiple sclerosis are infusional anti-CD20 drugs (Ocrelizumab and Rituximab), some of the most effective drugs currently available, which have been shown to reduce disease relapse by 46–47% annually [3]. The effects of these drugs do not appear to be limited only to the depletion of B lymphocytes; some studies illustrate how they may also act on other immune cells, such as NK cells, although this effect is still being studied [4]. Indeed, NK cells also appear to be involved in the control of the effector functions of T cells: the reduced immunoregulatory function of these cells could be one of the driving forces in the pathogenesis of autoimmune diseases, such as MS. To date, few studies have explored the effects of anti-CD20 drugs on NK cells in MS patients, and the available results are conflicting [4]. It has recently been found that some therapies used in MS, such as Natalizumab, Fingolimod, Interferon alpha, and autologous marrow transplantation, increase the number of NK cells [5]. In addition, therapies, including Glatiramer acetate, Vitamin D3, Dimethylfumarate, Monomethylfumarate, Natalizumab, Ocrelizumab, and IFN-β, have been shown to increase the biological activity of NK cells [6].

## 2. Results

We identified 64 patients, 37 females and 27 males, aged 25 to 72 years, with a mean of 51.6 ± 7.7 years (median: 52.0 years). Of these, 60 patients were on Ocrelizumab therapy, and 4 patients were on Rituximab therapy; 36 patients had relapsing remitting (RR) forms, and 28 patients were found to have progressive forms; the mean disease duration since diagnosis was 11.7 ± 8.1 years (median: 8.0 years). There were 16 patients naive from MS therapy. Of the 48 non-naive patients, 16 had previously used Natalizumab, 10/16 of whom received Natalizumab as a therapy immediately before starting Ocrelizumab/Rituximab (shift related to the risk of Progressive Multifocal Leukoencephalopathy secondary to JCV positivity); 10 patients were from Fingolimod. A total of 25 patients started the anti-CD20 drug for high disease activity coming from a first-line drug, while 10 patients came from a second-line drug and also had high disease activity. Three patients switched to Ocrelizumab due to intolerance/adverse effects of previous therapies. The cycles performed for single patients ranged from 2 to 8 (mean: 5.3 ± 2.2). EDSS at baseline ranged from 1 to 6 (mean: 3.7 ± 1.4), and EDSS before each therapy ranged from 1 to 7 (mean: 4.1 ± 1.4). Table 1 summarizes the characteristics of the sample. A total of 28 out of 298 urine culture tests performed 3 months after infusion were positive. Disease activity occurred in 97 of 277 total evaluations.

Initially, we analyzed the effect that the drug (Ocrelizumab/Rituximab) had on laboratory parameters assessed at 3 and 5 months compared with values at baseline (pre-infusion). These results are summarized in Table 2.

The data show that the absolute value of the concentration of T lymphocytes is reduced, but their percentage ratio to the overall number of lymphocytes increases, although not significantly. B lymphocyte levels are dramatically reduced (*p* < 0.001). The laboratory values that change significantly with therapy in both percentage and absolute terms are the levels of B lymphocytes (*p* < 0.001), which decrease, and NK cells, which increase (*p* < 0.001). Patients who already had high levels of NK cells before the start of the therapy did not show a greater tendency to increase NK cell levels after the infusion of the anti-CD20 drug (see the Table S1 in the Supplementary File).

We next assessed which individual characteristics or laboratory parameters, measured before starting the infusional anti-CD20 drug or 3 months after the first infusion could correlate with disease activity at the 6-month assessment after initiation. We then evaluated whether disease activity present on MRI before the initiation of therapy could correlate with disease activity assessed 6 months after anti-CD20 drug initiation. Table 3 summarizes the univariate analysis performed using baseline and laboratory data at 3 months compared with the assessment 6 months after the first course of therapy: only the EDSS value at baseline correlates with disease activity 6 months after the first infusion; indeed, the probability of finding active disease in the first 6 months after infusion correlates with EDSS (the higher the EDSS, the higher the probability of active disease, *p* = 0.044). No patients, either at initiation or 3 months after the first infusion, had hypogammaglobulinemia A.

Taking into consideration the low numerosity and the evaluation of only the first cycle of therapy reported in Table 2, we decided to extend our study to the evaluation of all cycles of Ocrelizumab/Rituximab performed, analyzing the characteristics of patients, type of disease, blood and urine tests performed 3 months after drug infusion compared with disease activity 6 months after therapy administration. Table 4 summarizes the univariate and multivariate analyses performed: from the univariate analysis on the sample, age, type of anti-CD20 infusion drug used, type of MS, pre-infusion EDSS, percentage of T cells and NK cells assessed 3 months after treatment correlated with the presence of disease activity 6 months after drug administration. However, on multivariate analysis, the only parameter that remains significant is the percentage level of natural killer cells 3 months after infusion.

In a total of 256 evaluations, early repopulation (i.e., found as early as 3 months after drug infusion of B lymphocytes) occurred in 29 cases. B lymphocyte repopulation 3 months after Ocrelizumab/Rituximab infusion was not shown to correlate with disease activity 6 months after drug infusion or with the immunoglobulin value or the presence/absence of hypogammaglobulinemia.

## 3. Discussion

When examining the pathogenesis of multiple sclerosis (MS), our primary focus centered on the well-established interplay between B and T lymphocytes. In particular, within the experimental autoimmune encephalomyelitis (EAE) model, B cells have been shown to interact with CD4+ T cells, initiating an adaptive immune response directed against myelin antigens. The inhibition of B-cell activity has yielded promising results, leading to reduced inflammation and improved clinical outcomes in EAE-afflicted rats [7]. In contrast, the role of natural killer (NK) cells in MS is marked by greater complexity and less clarity, although there is some evidence of interactions between B lymphocytes, T lymphocytes, and NK cells. Indeed, NK cells appear to be involved in the control of the effector functions of T cells [8]. Furthermore, NK cells, when co-cultured with B cells, can stimulate B cells to initiate polyclonal Ig secretion, and conversely, pre-activated B lymphocytes can induce NK cells to produce elevated levels of IFN-gamma [9]. However, tangible evidence for the pathogenetic involvement of NK cells in MS remains elusive and controversial, with some suggesting that NK cells ameliorate the disease, while others indicate their potential contribution to CNS damage [10].

The efficacy of disease-modifying therapies for multiple sclerosis relies on their ability to regulate not only the adaptive immune system but also the innate immune system, both of which play significant roles in the disease’s pathogenesis [8]. In addition to their effect on B lymphocytes, anti-CD20 antibodies are also known to act on other immune cells, such as T lymphocytes expressing the CD20 antigen on their cell surface [4]. Gingele et al. [11] have indeed shown that Ocrelizumab also reduces CD20+ T cells. However, it is still unclear whether and how Ocrelizumab affects the phenotype of those subsets of lymphocytes that do not express CD20, meaning almost all T lymphocytes and natural killer cells [4]. Numerous studies have endeavored to evaluate the influence of anti-CD20 treatments on natural killer cells; however, the currently available findings have yet to yield conclusive results. In our study, Ocrelizumab and Rituximab can result, as early as 3 months after the first infusion, in an increase in NK cells from baseline, associated with the known reduction in B lymphocytes. From the data presented by Abbadessa et al. [12] 6 and 12 months after infusion, there were no significant differences in the total absolute number of NK cells in MS patients treated with Ocrelizumab, while Landi et al. [13] even showed a decrease at 12 months in the absolute number of NK cells in MS patients treated with Ocrelizumab, in contrast to our data, which, however, assessed these values at 3 months. However, since the destructive action of anti-CD20 on B cells is linked to NK cell activation (antibody-dependent cellular cytotoxicity) [14], it is more logical to think that following the use of these drugs NK cells may increase.

Our data show that a lower percentage of NK 3 months after drug infusion correlates with disease activity 6 months after infusion; statistical significance, however, is lost when analyzing the absolute values of this cell population. No concordant or discordant evidence of these findings can be found in the literature. However, Wisgalla et al. [15] showed that percentage levels of NK cells increase during pregnancy of MS patients while decreasing significantly in the postpartum period [15]. Based on the assumption that pregnancy is protective against disease activity while postpartum increases the risk of MS reactivation [16], our data would be in line with the literature, confirming the protective effect of a higher percentage of NK cells on MS. Considering these data, we could confirm the hypothesis that NK cells play a role in the regulation of immunotolerance and thus in the pathogenesis of MS, as previously postulated [5], and that their alteration may indeed modify the course of the disease.

A further point to be analyzed concerns the protective effect of infusional anti-CD20 on MS: this could be related not only to their depletion action on B lymphocytes but could also be secondary to an increase in NK cells. The origin of this increase is not definitively understood; it remains uncertain whether it results from a direct impact of the drug on NK cells or if it is secondary to the activation of the Fc receptor on these cells (antibody-dependent cellular cytotoxicity), inducing a numerical expansion. If the first hypothesis were confirmed, then an additional question that will need to be addressed in the future is whether a greater percentage increase in NK cells may be more protective against disease activity. If, on the other hand, the second hypothesis were confirmed, then the evaluation of the expansion in NK cell levels after anti-CD20 drug therapy could potentially serve as a parameter for assessing the biological efficacy of anti-CD20 drugs.

In our study, the early repopulation of B lymphocytes 3 months after treatment did not predict disease activity 6 months after infusion. Other studies in the literature are in agreement with our data, suggesting that, contrary to what has been observed in neuromyelitis optica spectrum disorder (NMOSD), where there is a strong correlation between B-cell reappearance and disease exacerbation [17], such a correlation in MS does not exist. Indeed, a recent study found no significant difference in disease activity in MS patients treated with a fixed schedule of infusional anti-CD20 (every six months) compared with those treated with prolonged interval dosing (after B-cell reappearance). In addition, the authors did not find an association between CD19+ B-cell repopulation and disease activity [18]. This is likely since, even in patients who experience early B-cell repopulation, there is a recovery of B-cell subpopulations distinct from those present before the treatment. In contrast, Abbadessa et al. [12] evaluated lymphocyte repopulation and the effect on the disease after an interval longer than in our study by investigating B-cell numbers 6 months after infusion and disease activity at 12 months (evaluated only with MRI). It was observed in this study that patients in whom there is lymphocyte repopulation 6 months after treatment are more likely to have disease activity present on MRI 12 months after treatment than those who have slower repopulation; furthermore, in this study, patients with lymphocyte repopulation 6 months after Ocrelizumab infusion had higher B-cell counts at baseline than patients with late repopulation.

In our study, EDSS assessed before drug initiation is the only variable that can correlate with any disease activity 6 months after the first infusion of Ocrelizumab/Rituximab, in agreement with the findings of Cellerino et al. [19].

On the contrary, the serum immunoglobulin assay before therapy or 3 months after infusion and the presence/absence of hypogammaglobulinemia were not shown to correlate with disease activity 6 months after Ocrelizumab/Rituximab administration, a finding not currently found in the literature.

Six months after the initiation of infusion anti-CD20 therapy, no significant difference in disease activity was observed in patients put on Ocrelizumab/Rituximab therapy from Natalizumab, probably due to the high efficacy of the latter two therapies in reducing disease relapse. Similarly, patients administered Fingolimod did not have an increased risk of early disease activity, in contrast to the findings of Cellerino et al. [19].

Our study, limited to being single center and retrospective, aims to bring attention to a still under-explored topic, the role of NK cells in multiple sclerosis. Unfortunately, it was impossible to assay the different types of NK cells in CD56 bright, CD56 dim, and NKT cells. In the future, confirming our data on larger samples will be essential, possibly expanding the search to subcutaneous anti-CD20 drugs and studying different NK cell subtypes separately.

## 4. Materials and Methods

A retrospective single-center study was conducted on patients diagnosed with multiple sclerosis according to McDolnald’s 2017 criteria, referred to the Multiple Sclerosis Center of the Azienda Sanitaria Universitaria Friuli Centrale in Udine, Italy, on current or previous therapy with infusional anti-CD20 (Ocrelizumab or Rituximab). The data used were those available as of 31 December 2022. Patients with <2 cycles of therapy performed, patients who had an EDSS at the initiation of therapy >6, patients who had missed cycles of therapy, and patients who had started therapy in other multiple sclerosis centers for whom some data were unavailable were excluded. Administrative health laboratory and hospital discharge databases of the Udine ASUFC were used. The data are anonymized but linkable to each other at the individual patient level through a stochastic key, unique to each patient.

Ocrelizumab or Rituximab was administered according to FDA and EMA MS program approval: for Ocrelizumab, two 300 mg intravenous infusions two weeks apart, followed by 600 mg every six months; for Rituximab, two 1000 mg intravenous infusions two weeks apart, followed by 1000 mg every six months.

Blood tests, including white blood cells, neutrophils, lymphocytes, cytofluorimetry, IgG, IgA, and IgM levels; PCR; and urine culture tests were evaluated 3 and 5 months after drug infusion. Due to less-than-perfect adherence to the follow-up program, gathering all laboratory data for some patients was impossible. We defined “stable subjects” as those who presented 6 months after infusion with simultaneous subjectivity (including only new symptoms suggestive of disease relapse), objectivity (assessed on neurological objectivity and EDSS), MRI stability, and “disease activity” for all other patients, considering both relapse and progression. All primary progressive (PP), secondary progressive (SP), and relapsing progressive (RP) forms of MS were defined as progressive forms. To define the level of hypogammaglobulins, we took the reference values of IgG < 600 mg/dL, IgA < 40 mg/dL, and IgM < 70 mg/dL, as established in the literature [20,21]. Repopulation of B lymphocytes was defined if at least 0.1 B cell per µL was present. To define high levels of CD16+ NK cells before therapy, we used our laboratory’s cut-offs (>540 NK cells/µL in absolute value; >25% NK cells as a percentage value of total lymphocytes).

We evaluated the effect of Ocrelizumab/Rituximab on blood tests by comparing the results at baseline before starting therapy with subsequent assessments using the *t*-test for parametric variables and the Wilcoxon–Mann–Whitney test for nonparametric variables. To assess the presence of normal distribution, we used the Shapiro–Wilk normality test, using *p* < 0.05 as the cut-off. The calculated power of this study is 95%. We set an effect size of 0.039.

We used the chi-square test to investigate whether the presence of a high level of NK cells before the initiation of anti-CD20 drug therapy correlated with the expansion of the population of these cells on examinations 3 and 5 months after the first infusion.

We also used logistic regression (univariate and multivariate) to determine which variables studied could correlate with disease activity at 6 months. Odds ratio (OR) and 95% confidence intervals (95% CIs) were calculated. We considered *p* values < 0.05 to be statistically significant.

## 5. Conclusions

Ocrelizumab/Rituximab therapy, in addition to reducing B-cell counts, results in an increase in NK cells. Lower percentage levels of NK cells 3 months after anti-CD20 infusion correlate with the presence of disease activity 6 months after such therapy, suggesting a possible role of NK cells in the pathogenesis of MS. The levels of NK cells after anti-CD20 drug therapy could perhaps be used as a parameter to evaluate the biological efficacy of anti-CD20 drugs. The repopulation of B lymphocytes after 3 months has not been shown to correlate with disease activity 6 months after drug infusion. EDSS assessed before drug initiation is the only variable that correlates with any disease activity 6 months after the first Ocrelizumab/Rituximab infusion.

Further studies are needed to confirm or disprove our limited data.

## Figures and Tables

**Table 1:** Characteristics of the sample.

Variable	Mean ± SD or N° (%)
Number of patients	64
Age	51.6 ± 7.7 years
Female Sex	37 (57.8%)
Ocrelizumab therapy	60 (93.8%)
Disease duration	11.7 ± 8.1 years
Relapsing Remitting forms	36
EDSS at baseline	3.7 ± 1.4

**Table 2:** Effect of Ocrelizumab/Rituximab on blood tests at 3 and 5 months compared with pre-therapy tests.

Laboratory Data	Pre-Therapy Data (Mean ± DS)	Data 3 Months after First Infusion (Mean ± DS)	*p*	Pre-Therapy Data (Mean ± DS)	Data 5 Months after First Infusion (Mean ± DS)	*p*
**White blood cells (×10^3^/µL)**	6.136 ± 2.279	5.753 ± 1.389	0.472	6.136 ± 2.279	5.572 ± 1.465	0.150
**Neutrophils (×10^3^/µL)**	3.840 ± 1.788	3.566 ± 1.046	0.404	3.840 ± 1.788	3.575 ± 1.195	0.600
**Lymphocytes (×10^3^/µL)**	1.562 ± 0.794	1.389 ± 0.583	0.309	1.562 ± 0.794	1.268 ± 0.489	**<0.001**
**T lymphocytes (%)**	73.191 ± 8.618	81.816 ± 10.345	**<0.001**	72.975 ± 8.458	81.461 ± 9.010	**<0.001**
**Absolute T lymphocytes (/µL)**	1181.218 ± 681.857	1113.727 ± 491.025	0.580	1193.639 ± 657.038	1053.770 ± 426.021	0.120
**T Helper lymphocytes %**	48.653 ± 10.003	54.820 ± 11.824	**<0.001**	48.867 ± 9.960	54.779 ± 11.283	**<0.001**
**Absolute T Helper lymphocytes (/µL)**	800.927 ± 509.924	758.164 ± 376.959	0.738	813.131 ± 491.720	726.771 ± 354.866	0.150
**Cytotoxic T lymphocytes %**	22.604 ± 7.992	25.095 ± 9.074	**<0.001**	22.167 ± 7.775	24.956 ± 9.136	**<0.001**
**Absolute cytotoxic T lymphocytes (/µL)**	347.782 ± 238.687	335.309 ± 202.895	0.381	348.033 ± 231.441	308.246 ± 148.444	0.219
**CD4/CD8 ratio**	2.569 ± 1.401	2.591 ± 1.332	0.761	2.626 ± 1.446	2.613 ± 1.340	0.672
**NK cells %**	14.684 ± 7.716	18.243 ± 10.403	**<0.001**	14.567 ± 7.332	18.278 ± 9.301	**<0.001**
**Absolute NK cells (/µL)**	218.294 ± 144.328	245.961 ± 182.310	**<0.001**	220.621 ± 139.481	228.190 ± 136.194	**<0.001**
**B cells %**	12.278 ± 5.924	0.0398 ± 0.146	**<0.001**	12.331 ± 5.757	0.345 ± 0.836	**<0.001**
**Absolute B cells (/µL)**	213.922 ± 201.204	0.4821.636	**<0.001**	217.655 ± 199.695	4.386 ± 10.831	**<0.001**

**Table 3:** Univariate logistic regression to assess which demographic, clinical, and laboratory parameters at baseline and 3 months after the first anti-CD20 infusion correlate with disease activity 6 months after initiation of such therapy.

	Disease Activity 6 Months after the First Cycle		Univariate Analysis		
Variables	YES (24 Patients)	NO (40 Patients)			
	(N, %, Mean ± SD)	(N, %, Mean ± SD)	O.R.	CI (95%)	*p*
**Age (years)**	53.667 ± 7.637	50.300 ± 7.579	1.065	0.989–1.150	0.098
**Sex**	Females: 14/24, Males: 10/24	Females: 23/40, Males: 17/40	0.966	0.347–2.690	0.966
**Ocrelizumab/Rituximab**	Ocrelizumab: 20/24, Rituximab: 4/24	Ocrelizumab: 40/40, Rituximab: 0/40	8.51 × 10^7^	0.000-inf	0.993
**EDSS at baseline**	4.125 ± 1.304	3.400 ± 1.345	1.528	1.012–2.310	**0.044**
**Years since diagnosis**	12.083 ± 7.723	11.425 ± 8.500	1.010	0.949–1.070	0.753
**Previous therapies**	No: 6/24, Yes: 18/24	No: 10/40, Yes: 30/40	1.000	0.311–3.220	1.000
**Type of Disease**	Inflammatory forms: 12/24, Progressive forms: 12/24	Inflammatory forms: 24/40, Progressive forms: 16/40	1.500	0.541–4.160	0.436
**Pre-treatment MRI activity**	No: 10/22 Yes: 12/22	No: 20/40 Yes: 20/40	1.200	0.423–3.406	0.732
**Prior use of Natalizumab**	No: 18/24, Yes: 6/24	No: 31/40, Yes: 9/40	1.148	0.351–3.760	0.819
**Natalizumab as the last therapy**	No: 21/24, Yes: 3/24	No: 33/40, Yes: 7/40	0.673	0.157–2.900	0.595
**Fingolimod as the last therapy**	No: 20/24, Yes: 4/24	No: 34/40, Yes: 6/40	1.133	0.285–4.510	0.859
**White blood cells at baseline (×10^3^/µL)**	6.423 ± 3.188	5.964 ± 1.521	1.093	0.870–1.370	0.446
**Neutrophils at baseline (×10^3^/µL)**	4.169 ± 2.578	3.642 ± 1.066	1.190	0.861–1.640	0.292
**Lymphocytes at baseline (×10^3^/µL)**	1.514 ± 0.751	1.591 ± 0.827	0.883	0.461–1.690	0.708
**T lymphocytes at baseline (%)**	75.325 ± 6.587	71.597 ± 9.162	1.059	0.991–1.130	0.093
**Absolute T lymphocytes at baseline (/µL)**	1206.167 ± 724.311	1169.158 ± 619.621	1.000	0.999–1.000	0.828
**T Helper lymphocytes at baseline (%)**	49.546 ± 9.479	48.363 ± 10.231	1.012	0.9610–1.070	0.644
**Absolute T Helper lymphocytes at baseline (/µL)**	822.000 ± 574.490	795.000 ± 438.940	1.000	0.999–1.000	0.832
**Cytotoxic T lymphocytes at baseline (%)**	23.583 ± 8.518	21.453 ± 7.235	1.037	0.969–1.110	0.292
**Absolute cytotoxic T lymphocytes at baseline (/µL)**	360.333 ± 241.821	336.684 ± 225.346	1.000	0.998–1.000	0.692
**CD4/CD8 ratio at baseline**	2.558 ± 1.563	2.642 ± 1.378	0.959	0.668–1.380	0.822
**NK cells at baseline (%)**	12.786 ± 6.104	15.422 ± 7.892	0.949	0.878–1.030	0.185
**Absolute NK cells at baseline (/µL)**	194.682 ± 112.730	231.459 ± 153.812	0.998	0.994–1.000	0.331
**B cells at baseline (%)**	11.464 ± 6.178	12.946 ± 5.454	0.953	0.863–1.050	0.337
**Absolute B cells at baseline (/µL)**	206.545 ± 227.094	221.541 ± 182.338	1.000	0.997–1.000	0.777
**IgG at baseline**	1009.261 ± 221.881	931.286 ± 239.363	1.002	0.999–1.000	0.218
**IgG reduced at baseline**	No: 23/23, Yes: 0/23	No: 33/35, Yes: 2/35	9.17 × 10^−8^	0.000-inf	0.992
**IgA at baseline**	189.391 ± 67.448	228.829 ± 167.459	0.997	0.991–1.000	0.312
**IgA reduced at baseline**	No: 23/23, Yes: 0/23	No: 35/35, Yes: 0/35	NA	NA	NA
**IgM at baseline**	90.348 ± 49.247	100.571 ± 52.520	0.996	0.985–1.010	0.455
**IgM reduced at baseline**	No: 15/23, Yes: 8/23	No: 24/35, Yes: 11/35	1.164	0.381–3.550	0.790
**PCR at baseline**	2.455 ± 3.586	2.073 ± 3.260	1.034	0.891–1.200	0.659
**Urine culture test at baseline**	Negative: 24/24, Positive: 0/24	Negative: 37/40, Positive: 3/40	9.85 × 10^−8^	0.000-inf	0.991
**White blood cells at 3 months (×10^3^/µL)**	6.051 ± 1.365	5.574 ± 1.389	1.289	0.885–1.880	0.187
**Neutrophils at 3 months (×10^3^/µL)**	3.825 ± 0.940	3.410 ± 1.087	1.481	0.894–2.455	0.128
**Lymphocytes at 3 months (×10^3^/µL)**	1.408 ± 0.646	1.378 ± 0.549	1.092	0.458–2.600	0.843
**T lymphocytes at 3 months (%)**	83.757 ± 9.612	80.778 ± 10.447	1.031	0.974–1.090	0.287
**Absolute T lymphocytes at 3 months (/µL)**	1101.000 ± 505.869	1101.000 ± 477.713	1.000	0.999–1.000	0.947
**T Helper lymphocytes at 3 months (%)**	54.252 ± 12.370	54.606 ± 12.370	0.998	0.953–1.040	0.914
**Absolute T Helper lymphocytes at 3 months (/µL)**	719.762 ± 337.697	765.250 ± 397.681	1.000	0.998–1.000	0.656
**Cytotoxic T lymphocytes at 3 months (%)**	27.262 ± 9.021	24.519 ± 9.752	1.031	0.974–1.090	0.294
**Absolute cytotoxic T lymphocytes at 3 months (/µL)**	365.238 ± 272.243	322.389 ± 149.505	1.001	0.998–1.000	0.449
**CD4/CD8 ratio at 3 months**	2.343 ± 1.270	2.673 ± 1.374	0.821	0.535–1.260	0.368
**NK cells at 3 months (%)**	15.652 ± 9.089	19.334 ± 10.397	0.960	0.905–1.020	0.186
**Absolute NK cells at 3 months (/µL)**	210.857 ± 181.425	259.229 ± 171.962	0.998	0.995–1.000	0.321
**B cells at 3 months (%)**	0.063 ± 0.218	0.020 ± 0.053	13.134	0.059–2913.613	0.350
**Absolute B cells at 3 months (/µL)**	0.757 ± 2.407	0.249 ± 0.676	1.270	0.790–2.040	0.324
**B-lymphocyte repopulation at 3 months**	No: 16/21, Yes: 5/21	No: 30/35, Yes: 5/35	1.875	0.472–7.454	0.372
**IgG at 3 months**	975.435 ± 182.352	997.029 ± 222.740	0.999	0.997–1.000	0.696
**IgG reduced at 3 months**	No: 23/23, Yes: 0/23	No: 33/34, Yes: 1/34	2.49 × 10^−7^	0.000-inf	0.992
**IgA at 3 months**	194.652 ± 65.610	243.176 ± 231.569	0.998	0.993–1.000	0.374
**IgA reduced at 3 months**	No: 23/23, Yes: 0/23	No: 34/34, Yes: 0/34	NA	NA	NA
**IgM at 3 months**	77.348 ± 48.256	89.088 ± 52.889	0.995	0.984–1.010	0.393
**IgM reduced at 3 months**	No: 10/23, Yes: 13/23	No: 21/34, Yes: 13/34	2.100	0.716–6.160	0.177
**PCR at 3 months**	2.480 ± 4.080	1.395 ± 3.001	1.097	0.934–1.288	0.260
**Urine culture test at 3 months**	Negative: 22/23, Positive: 1/23	Negative: 31/39, Positive: 8/39	0.176	0.021–1.510	0.113

**Table 4:** Univariate and multivariate logistic regression evaluating which demographic, clinical, and laboratory parameters 3 months after anti-CD20 infusion correlate with disease activity assessed 6 months after such therapy.

	Disease Activity 6 Months after Infusion		Univariate Analysis			Multivariate Analysis		
Variables	YES (97 Evaluations)	NO (180 Evaluations)						
	(N, %, Mean ± SD)	(N, %, Mean ± SD)	O.R.	CI (95%)	*p*	O.R.	CI (95%)	*p*
**Age (years)**	53.979 ± 7.264	51.500 ± 7.496	1.047	1.011–1.084	**0.010**	1.030	0.986–1.075	0.185
**Sex**	Males: 40/97, Females: 57/97	Males: 88/180, Females: 92/180	0.734	0.446–1.208	0.224			
**Ocrelizumab/Rituximab**	Ocrelizumab: 84/97, Rituximab: 13/97	Ocrelizumab: 175/180, Rituximab: 5/180	5.417	1.870–15.693	**0.002**	2.547	0.719–9.026	0.148
**Type of disease**	Inflammatory forms: 45/97, Progressive forms: 52/97	Inflammatory forms: 114/180, Progressive forms: 66/180	1.996	1.209–3.295	**0.007**	1.130	0.601–2.124	0.704
**Years since diagnosis**	11.794 ± 7.896	13.033 ± 8.235	0.981	0.951–1.011	0.226			
**Pre-infusion EDSS**	4.490 ± 1.205	3.906 ± 1.374	1.405	1.154–1.709	**<0.001**	1.230	0.969–1.560	0.089
**White blood cells at 3 months (×10^3^/µL)**	6.104 ± 1.475	5.849 ± 1.575	1.111	0.948–1.303	0.193			
**Neutrophils at 3 months (×10^3^/µL)**	3.887 ± 1.176	3.743 ± 1.391	1.085	0.902–1.305	0.387			
**Lymphocytes at 3 months (×10^3^/µL)**	1.406 ± 0.517	1.313 ± 0.498	1.432	0.883–2.322	0.145			
**T lymphocytes at 3 months (%)**	83.274 ± 8.669	80.750 ± 9.723	1.030	1.001–1.060	**0.041**	0.601	0.359–1.006	0.053
**Absolute T lymphocytes at 3 months (/µL)**	1149.337 ± 439.102	1056.735 ± 433.407	1.000	0.999–1.001	0.104			
**T Helper lymphocytes at 3 months (%)**	54.554 ± 11.584	53.021 ± 11.489	1.012	0.989–1.035	0.306			
**Absolute T Helper lymphocytes at 3 months (/µL)**	758.402 ± 331.842	702.313 ± 335.241	1.000	0.999–1.001	0.198			
**Cytotoxic T lymphocytes at 3 months (%)**	27.111 ± 9.176	26.137 ± 9.739	1.011	0.984–1.038	0.432			
**Absolute cytotoxic T lymphocytes at 3 months (/µL)**	374.011 ± 215.725	333.163 ± 166.193	1.001	0.999–1.002	0.096			
**CD4/CD8 ratio at 3 months**	2.359 ± 1.202	2.403 ± 1.212	0.969	0.784–1.199	0.776			
**NK cells at 3 months (%)**	16.399 ± 8.521	19.141 ± 9.692	0.968	0.940–0.996	**0.026**	0.582	0.346–0.978	**0.041**
**Absolute NK cells at 3 months (/µL)**	221.196 ± 114.926	246.598 ± 153.956	0.999	0.997–1.001	0.195			
**B cells at 3 months (%)**	0.021 ± 0.108	0.020 ± 0.090	1.075	0.775–14.896	0.957			
**Absolute B cells at 3 months (/µL)**	0.258 ± 1.216	0.213 ± 0.823	1.046	0.812–1.347	0.730			
**B-lymphocyte repopulation at 3 months**	Yes: 10/92, No: 82/92	Yes: 19/164, No: 145/164	0.931	0.413–2.097	0.862			
**IgG at 3 months**	902.341 ± 213.151	930.671 ± 230.599	0.999	0.998–1.001	0.337			
**IgG reduced at 3 months**	Yes: 3/91, No: 88/91	Yes: 8/158, No: 150/158	0.639	0.165–2.472	0.517			
**IgA at 3 months**	206.506 ± 209.442	241.840 ± 251.377	0.999	0.998–1.001	0.275			
**IgA reduced at 3 months**	Yes: 2/97, No: 95/97	Yes: 2/156, No: 154/156	1.730	0.239–12.498	0.587			
**IgM at 3 months**	64.156 ± 44.936	65.459 ± 42.164	0.999	0.993–1.005	0.819			
**IgM reduced at 3 months**	Yes: 63/90, No: 27/90	Yes: 97/157, No: 60/157	1.443	0.829–2.511	0.194			
**PCR at 3 months**	2.017 ± 3.176	2.274 ± 6.474	0.991	0.941–1.043	0.720			
**Urine culture test at 3 months**	Positive: 9/94, Negative: 85/94	Positive: 18/178, Negative: 160/178	0.941	0.405–2.185	0.888			

## Data Availability

Data is contained within the article and Supplementary Material.

## Supplementary Materials

The following supporting information can be downloaded at: https://www.mdpi.com/article/10.3390/ph17020150/s1, Table S1: The table examine whether the presence of a high level of NK cells before the start of anti-CD20 drug therapy correlates with the expansion of the population of these cells on examination at 3 and 5 months after the first infusion.
