# Supplementary material for: NK Cell Levels Correlate with Disease Activity in Patients with Multiple Sclerosis on Ocrelizumab/Rituximab Therapy
Source: Pharmaceuticals (Basel). 2024 Jan 23;17(2):150. doi: 10.3390/ph17020150 (PMC10891999; doi:10.3390/ph17020150)
Supplement: Supplementary file 1 [file pharmaceuticals-17-00150-s001.zip › pharmaceuticals-2820881-supplementary.pdf]

Table 1. The table examine whether the presence of a high level of NK cells before the start of anti-CD20 drug therapy correlates with the expansion of the population of these cells on examination at 3 and 5 months after the first infusion.

	**Pre-therapy NK cells > 540 /µL**		
**Variables**	**No**	**Yes**	***p***
**Expansion of NK cells in absolute value 3 months after initiation of therapy**	No 22/47<br>Yes 25/47	No 2/3<br>Yes 1/3	0.504
**Expansion of NK cells in percent value 3 months after initiation of therapy**	No 17/48<br>Yes 31/48	No 1/3<br>Yes 2/3	0.942
**Expansion of NK cells in absolute value 5 months after initiation of therapy**	No 18/47<br>Yes 29/47	No 3/3<br>Yes 0/3	**0.036**
**Expansion of NK cells in percent value 5 months after initiation of therapy**	No 13/47<br>Yes 34/47	No 1/3<br>Yes 2/3	0.832
	**Pre-therapy NK cells > 25%**		
**Variables**	**No**	**Yes**	***p***
**Expansion of NK cells in absolute value 3 months after initiation of therapy**	No 23/45<br>Yes 22/45	No 1/5<br>Yes 4/5	0.187
**Expansion of NK cells in percent value 3 months after initiation of therapy**	No 16/46<br>Yes 30/46	No 2/5<br>Yes 3/5	0.817
**Expansion of NK cells in absolute value 5 months after initiation of therapy**	No 20/45<br>Yes 25/45	No 1/5<br>Yes 4/5	0.293
**Expansion of NK cells in percent value 5 months after initiation of therapy**	No 13/45<br>Yes 32/45	No 1/5<br>Yes 4/5	0.675
